# Recanalization of Xen45 gel stent implant occlusion using 10 − 0 nylon suture in refractory glaucoma: a case report

**DOI:** 10.1186/s12886-023-03109-7

**Published:** 2023-10-19

**Authors:** Yao Zhang, Haotian Xiang, Yangyang Zhang, Li Tang

**Affiliations:** Department of Ophthalmology, West China Hospital of Sichuan University, Chengdu, China

**Keywords:** Glaucoma, Micro-invasive glaucoma surgery, Occlusion, Postoperative complications, Recanalization, Xen gel stent

## Abstract

**Background:**

Xen Gel Stent implant is a new minimally invasive surgical treatment for glaucoma that has been proven effectiveness and safety profile. However, it may also lead to some complications. Xen Gel Stent occlusion is a relatively rare complication reported less frequently and has limited treatment experience. In our case report, we proposed a novel surgical treatment using a 10 − 0 nylon suture to successfully recanalize the occluded Xen45 Gel Stent.

**Case presentation:**

A 16-year-old female patient had bilateral juvenile glaucoma for the past 5 years. Her right eye had undergone three glaucoma surgeries but failed. At a presentation to our hospital, the right eye’s intraocular pressure (IOP) was 30 mmHg despite applying four different active principles. Xen45 Gel Stent implant was chosen for treatment, but six days after implantation, the IOP rose to 40 mmHg as a result of an anterior chamber tip occlusion of the Xen45 Gel Stent. Nd: YAG laser shockwave therapy was attempted but failed. The patient eventually had to return to the operating room for a revision procedure. The Xen45 Gel Stent was recanalized from the ab externo by making an L-shaped conjunctival incision at the fornix base and threading a 10 − 0 nylon suture through it. The IOP was successfully controlled in the 11-month follow-up without medication.

**Conclusion:**

If postoperative occlusion arises after Xen45 Gel Stent implantation, surgery using 10 − 0 nylon suture to recanalize Xen45 Gel Stent should be considered as a relatively safe, effective that does not require removal of Xen45 Gel Stent.

**Supplementary Information:**

The online version contains supplementary material available at 10.1186/s12886-023-03109-7.

## Background

Glaucoma is a leading cause of irreversible blindness worldwide. According to recent estimates, the global prevalence of the disease would approach 111 million cases by 2040 [1]. Although trabeculectomy surgery remains the gold standard for glaucoma treatment, it comes with numerous risks [2].

Xen45 Gel Stent (Allergan-Abbvie, California, USA)-a 6-mm-long tubular implant with a 45-µm inner lumen, is the only device that mimics the nonphysiologic drainage pathway of trabeculectomy and aqueous shunt surgeries while obviating the need for conjunctival dissection [3]. Xen45 Gel Stent implant is effective and safe in the treatment of open-angle glaucoma, including primary and secondary glaucoma [4–8]. Combined with phacoemulsification, it can also achieve similar effects in treating angle-closure glaucoma [8–9]. Although Xen45 Gel Stent has the potential to achieve a lower final IOP, it comes with a higher complication rate and requires rigorous postoperative monitoring [10]. Transient hypotony, hyphema, choroidal effusion, and corneal endothelia cell loss are common complications but usually transient and self-limited [11–12]. Other relatively rare complications include implant exposure, implant malposition, and implant occlusion requiring neodymium-doped yttrium aluminum garnet (Nd: YAG) laser shock wave treatment or surgical revision [5, 6, 8, 13, 14].

Herein, we describe a case of Xen45 Gel Stent used for the treatment of refractory glaucoma and propose a new surgical protocol to recanalize the occluded Xen45 Gel Stent using a 10 − 0 nylon suture successfully without removal of Xen45 Gel Stent.

## Case presentation

### History

A 16-year-old female patient presented at our clinic with poorly controlled IOP in the right eye. She had been diagnosed with bilateral juvenile open-angle glaucoma (JOAG) and underwent gonioscopy-assisted transluminal trabeculotomy (GATT) in both eyes 4 years earlier, but the IOP in her right eye rose again one year after the surgery. Subsequently, goniosynechialysis (GSL) and trabeculectomy were performed in the right eye. There was no known family history of glaucoma.

On examination, the best-corrected visual acuity (BCVA) was hand-motion in the right eye and 0.02 in the left eye. The IOP was 33 mmHg in the right eye on 4 antiglaucoma medications and 13 mmHg in the left eye on 1 antiglaucoma medication. Slit-lamp examination revealed a flattened superior bleb, a recognizable peripheral iridectomy, a moderately deep anterior chamber, and a transparent lens in both eyes. Fundus examination revealed bilateral glaucomatous optic disc with a cup-to-disc ratio of 1.0. Gonioscopy showed opened schlemm’s canal and some peripheral anterior synechiae at 8 clock points.

## Xen45 gel stent implantation

**Fig. 1:**
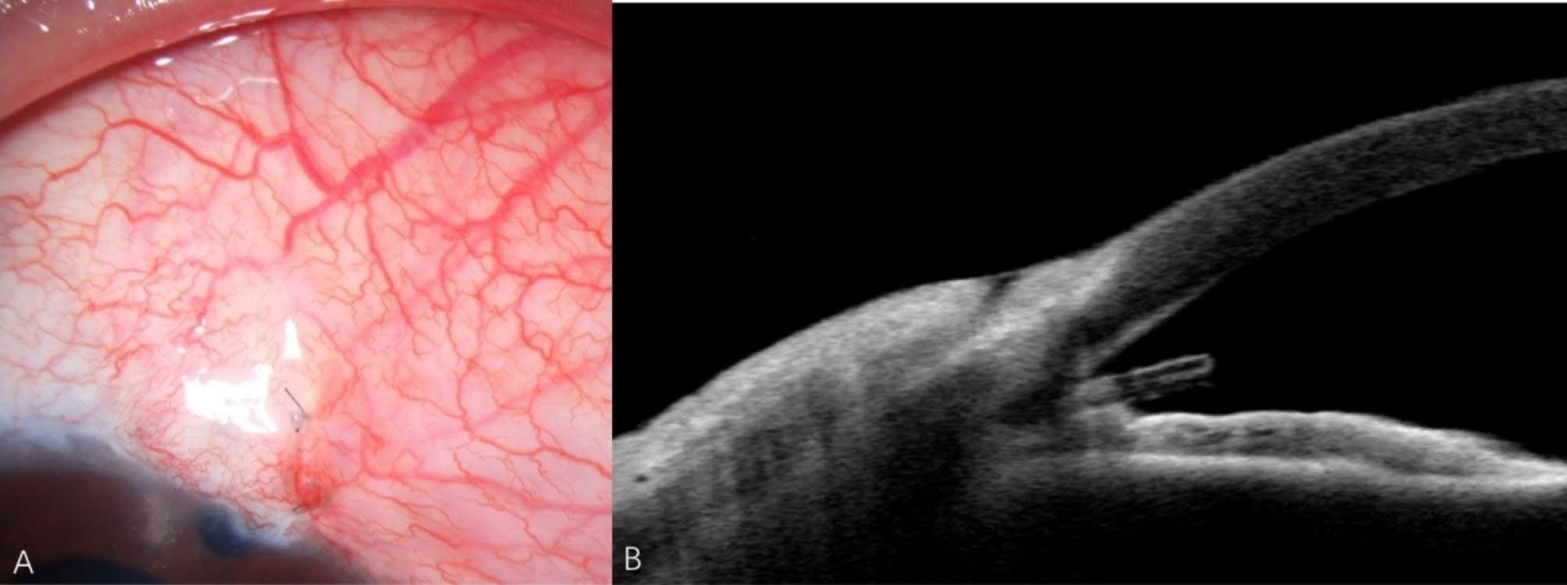
(A) Photographs of anterior segment showed a diffuse filtering bleb. (B) The AS-OCT showed the internal ostium was clear

The Xen45 Gel Stent was implanted in the right eye at the 1 o’clock position after subconjunctival injection of mitomycin-C (0.1ml, 0.02%). Antibiotics and steroids eye drops were applied 4 times a day after surgery. The IOP decreased to 5 mmHg with a choroidal detachment on the first postoperative day, which disappeared after 5 days. The IOP stabilized at 10 mmHg with a diffuse filtering bleb. The AS-OCT (AngioVue®, Optovue, Fremont, CA) showed the inner of Xen45 Gel Stent was clear (Fig. 1).

**Fig. 2:**
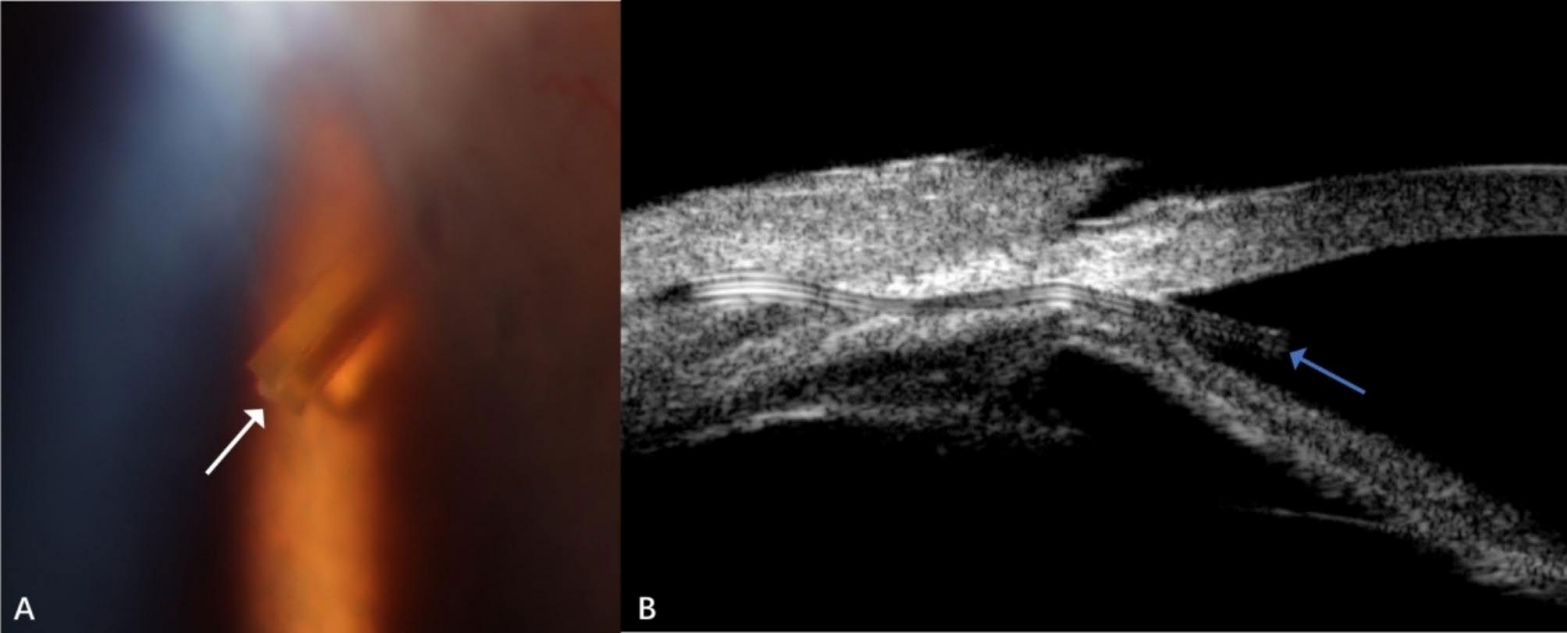
(A) The anterior segment examination showed a tiny whitish plug (white arrow) occluded the anterior chamber tip of Xen45 Gel Stent. (B) The UBM showed the hyper-reflective materials (blue arrow) at the internal ostium of the stent

Six days after surgery, the IOP spiked to 40 mmHg, and a tiny droplet of whitish material occluded the anterior chamber tip of Xen45 Gel Stent was found. Ultrasound biomicroscopy (UBM)(SW-3200 L, Suoer, China) revealed the hyper-reflective material in the internal ostium of Xen45 Gel Stent (Fig. 2).

## Nd: YAG laser shockwave treatment

**Fig. 3:**
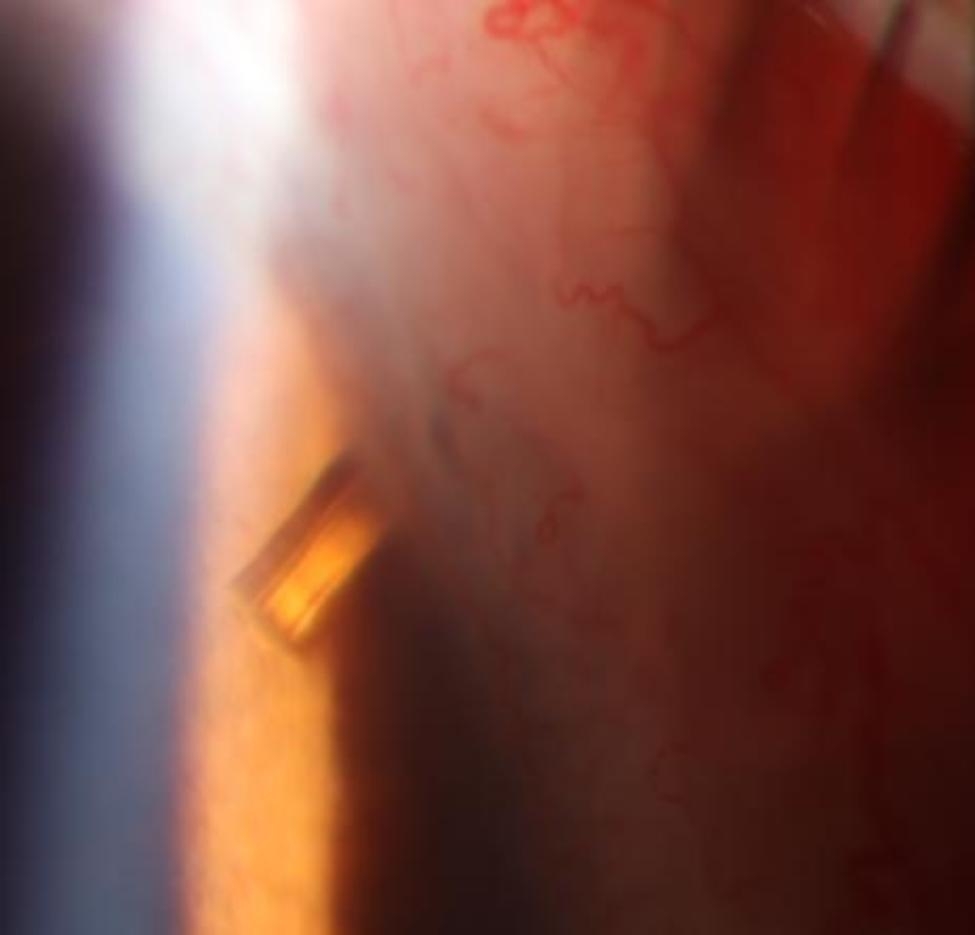
Slit-lamp examination revealed the inner opening of Xen Gel Stent was clear after Nd:YAG laser shockwave treatment

Nd: YAG laser ( Selecta Duet, Lumenis, USA ) shockwave therapy was conducted with 0.4 mJ, 6 pulses directed just anterior to the tip. We could see some bubbles rising from the tip of the tube lumen. After treatment, the internal ostium of Xen was visible on slit-lamp examination (Fig. 3). The IOP decreased from 42 to 25 mmHg.

However, only 6 h after the laser therapy, the IOP elevated to 40mmHg again. Several Nd: YAG lasers attempted, with energy increasing from 0.4 mJ to 1.0 mJ, failed. The slit lamp showed that the blockage was invisible, but the IOP remained significantly elevated.

## Recanalization of Xen45 gel stent using 10 − 0 nylon suture

An L-shaped conjunctival incision was made at the fornix base (10–12 o’clock). After removing the scar tissue around Xen45 Gel Stent, no outflow of aqueous humor was observed. A 10 − 0 nylon suture (W1770, Ethicon, Johnson, USA) was probed from the conjunctival ostium of Xen45 Gel Stent and threaded into the anterior chamber segment under gonioscopic guidance. After removing the 10 − 0 nylon suture, the outflow of aqueous humor immediately improved, and the occlusion seemed to be resolved. The conjunctival flap was then sutured and mitomycin-C (0.1ml, 0.02%) was injected subconjunctivally (Additional file 1).

**Fig. 4:**
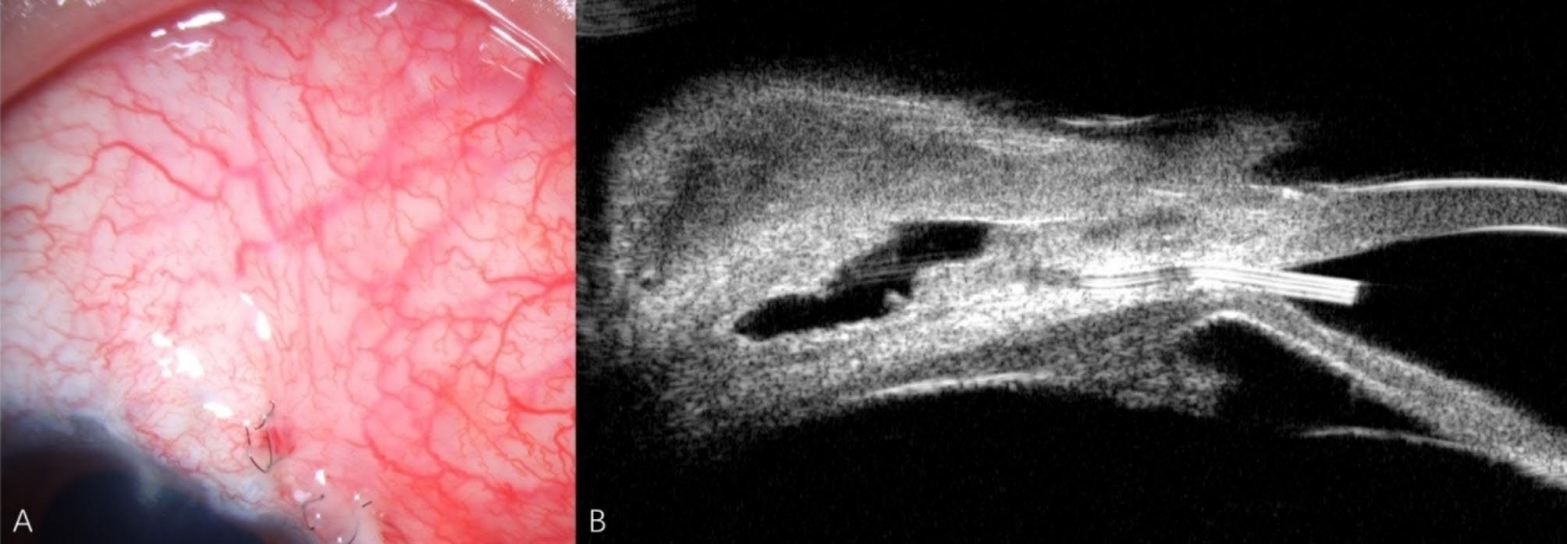
The anterior segment examination (A) and UBM (B) revealed a functioning filtering bleb at 1 day postoperatively

The IOP was 8 mmHg, and examination of the anterior segment and UBM revealed a functioning filtering bleb at 1 day (Fig. 4). Postoperatively, topical antibiotics and corticosteroid drops were provided, with the dose gradually decreasing over a month. The IOP was 10 mmHg at 1 week, stabilizing at 12 mmHg at 1, 3, and 6 months. The IOP was 15 mmHg at 11 months postoperative visit without medication.

## Discussion and conclusions

The aim of this report is two-fold: first, to present a case of Xen45 Gel Stent for the treatment of refractory glaucoma; and second, to propose a new solution to successfully recanalize the Xen45 Gel Stent using 10 − 0 nylon sutures. There are few studies on Xen45 Gel Stent on individuals with refractory glaucoma who have had failed glaucoma filtration procedures [15]. In our case, the patient suffered from uncontrolled IOP despite maximum tolerated antiglaucoma drug treatment and previous multiple unsuccessful antiglaucoma surgeries (GATT and trabeculectomy). The patient is so young that the filtering bleb is prone to scarring, which leads to surgical failure. The safety of Xen Gel Stent implantation in terms of complications appears to be better than conventional glaucoma surgery [16]. The Xen Gel Stent is implanted through an ab-interno ‘closed conjunctiva’ method and drains aqueous fluid to the subconjunctival region without the use of an extraocular reservoir, if Xen implantation fails, it is almost possible to rescue the Xen45 with the apposition of performing a filtering surgery as deep sclerectomy or trabeculectomy, adding a Baerveldt or another glaucoma drainage device [17–21]. Moreover, at the time of development, three different Xen models, with different lumen sizes were created: 45 μm (Xen45), 63 μm (Xen63), and 140 μm (Xen140). However, initially, only Xen45 was made commercially available. Xen45 has previously been shown to produce effective IOP lowering with a good safety profile [22]. In the future, we can try to use the novel Xen63 or other models to compare with Xen45 device.

In the meta-analysis by Xz et al. [13], the common postoperative complications after Xen implantation were transient hypotony(9.59%), hyphema (5.53), transient IOP spikes ≥ 30 mmHg (2.11% on average), while implant occlusion (0.93%), implant malposition (0.88%), and implant exposure(0.57%) were relatively uncommon. Fellman et al. [23] reported six patients with intraluminal cellular debris after Xen45 surgery were successfully treated with low energy Nd: YAG laser shockwave therapy aimed just the intracameral tip of the gel stent through a gonioscopy lens. Seo et al. [24] used the Nd: YAG laser (0.8 mJ, 2 shots) to successfully recanalize an Xen45 Gel Stent occlusion with cortical material.

In our case, the probable materials were fibrin plugs or cellular debris, which could not be clearly diagnosed histologically. The failure of laser treatment was most likely due to the blocking of the distal end of the Xen45 Gel Stent, such as the scleral or conjunctival section, and the laser energy was insufficient to disperse the obstruction. This is the first time we tried Nd: YAG laser treatment for Xen45 Gel Stent occlusion. The choice of laser energy level is still in the exploratory stage. More evidence is needed to verify whether a higher laser energy level will damage the tube or be able to disperse the more distal occlusion.

Pinto Ferriera et al. [25] used a 23-G inner limiting membrane forceps to remove the blood clot over the ostium. Tatti et al. [26] reported a case in which a 25 G vitreous scissors were used to trim ab interno of Xen45 Gel Stent occluded by the high adhesiveness of the fibrin clot after a failed Nd: YAG laser treatment. In our case, a 10 − 0 nylon suture was utilized to recanalize the Xen45 Gel Stent occlusion by intraluminal cellular debris or fibrin. The interior of the lumen was not damaged by 10 − 0 nylon suture and the IOP was well controlled at the 11 months follow-up after surgery without the need for antiglaucoma drugs or a further second Xen device. To the best of our knowledge, this is the first case report of recanalization of Xen45 Gel Stent occlusion using the 10 − 0 nylon suture therapy.

The inner diameters of Xen45 Gel Stent were designed considering the total implant length, viscosity of aqueous humor, and typical aqueous production rates of the human eye [27]. The Xen45, with the smallest inner lumen (45 μm), can maximize long-term outflow and maintain IOP around 6–8 mmHg to prevent hypotony [28], but it also has a risk of occlusion. The exact causes of occlusion are still unknown, but postoperative anterior chamber bleed resulting in the occlusion of red blood cells or their by-products is one possibility [29]. Another potential cause is fibrin formation due to chronic intraocular inflammation [30]. Cortical material can also contribute to occlusion after combined phacoemulsification and Xen implantation [24].

Xen45 Gel Stent implant is a novel surgical treatment that has demonstrated both efficacy and safety, despite having relatively high rates of needling and reoperations [31]. However, due to its recent introduction, there is a limited amount of experience in performing the surgical procedure and managing any potential complications that may arise. Our case highlights the importance of controlling inflammation both preoperatively and postoperatively, as well as preventing bleeding during surgery, in order to reduce the risk of occlusion of Xen45 Gel Stent.

If postoperative occlusion arises after Xen45 Gel Stent implantation, Nd: YAG laser shockwave should be tried first due to its minimal invasiveness, and if that fails, surgery with 10 − 0 nylon suture to recanalize Xen45 gel stent should be considered as a relatively safe, effective, and alternative that could prevent from removing of Xen45 Gel Stent. These strategies can help increase the success rate of Xen45 Gel Stent implantation.

### Electronic supplementary material

Below is the link to the electronic supplementary material.

Supplementary material 1: A surgical video of recanalization of Xen45 Gel Stent

Supplementary material 2: Timeline of treatment and outcome

## Data Availability

All data generated or analyzed during this study are included in this published article [and its supplementary information files].
