# Supplementary figures and images for: Recanalization of Xen45 gel stent implant occlusion using 10 − 0 nylon suture in refractory glaucoma: a case report
Source: BMC Ophthalmol. 2023 Oct 19;23:418. doi: 10.1186/s12886-023-03109-7 (PMC10585744; doi:10.1186/s12886-023-03109-7)

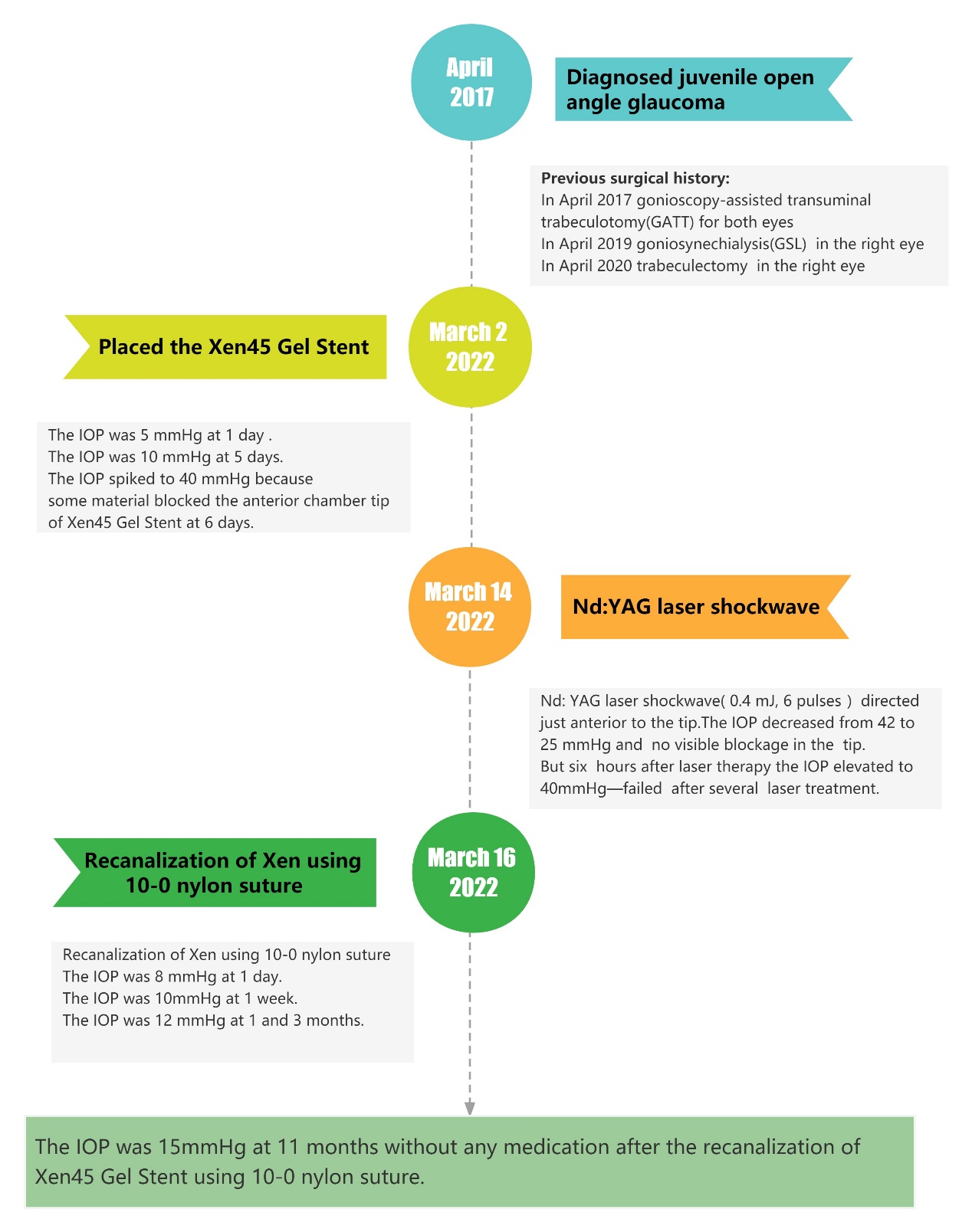


Additional file 2. Timeline of treatment and outcome

Supplement: Supplementary file 2 — Supplementary material 2: Timeline of treatment and outcome [file 12886_2023_3109_MOESM2_ESM.docx]
